# Syntheses, crystal structures, Hirshfeld surface analyses and energy frameworks of two 4-amino­anti­pyrine Schiff base compounds: (*E*)-4-{[4-(di­ethyl­amino)­benzyl­idene]amino}-1,5-dimethyl-2-phenyl-1*H*-pyrazol-3(2*H*)-one and (*E*)-4-[(4-fluoro­benzyl­idene)amino]-1,5-dimethyl-2-phenyl-1*H*-pyrazol-3(2*H*)-one

**DOI:** 10.1107/S2056989023004085

**Published:** 2023-05-12

**Authors:** M. G. Shankar, R. Kumaravel, A. Subashini, K. Ramamurthi, Monika Kučeráková, Michal Dušek, Helen Stoeckli-Evans

**Affiliations:** a PG and Research Department of Physics, Srimad Andavan Arts Science College (Autonomous), Affiliated to Bharathidasan University, Tiruchirappalli - 620 005, Tamilnadu, India; b Department of Physics, Annapoorana Engineering College, Salem – 636 308, Tamilnadu, India; c Crystal Growth and Thin Film Laboratory, Department of Physics, Bharathidasan University, Tiruchirappalli - 620024, Tamilnadu, India; d Institute of Physics ASCR, Na Slovance 2, 182 21 Praha 8, Czech Republic; e Institute of Physics, University of Neuchâtel, rue Emile-Argand 11, 2000 Neuchâtel, Switzerland; University of Aberdeen, United Kingdom

**Keywords:** crystal structure, 4-amino­anti­pyrine, Hirshfeld surface analysis, energy frameworks

## Abstract

The title Schiff base compounds, C_22_H_26_N_4_O (**I**) and C_18_H_16_FN_3_O (**II**), were each synthesized by a single-step condensation reaction. The substituted benzyl­idene ring is inclined to the pyrazole ring mean planes by 22.92 (7)° in **I** and 12.70 (9)° in **II**. The phenyl ring of the 4-amino­anti­pyrine unit is inclined to the pyrazole ring mean plane by 54.87 (7)° in **I** and by 60.44 (8)° in **II**. In the crystal of **I**, the mol­ecules are linked by C—H⋯O hydrogen bonds and C—H⋯π inter­actions to form layers lying parallel to (001). In the crystal of **II**, the mol­ecules are linked by C—H⋯O and C—H⋯F hydrogen bonds and C—H⋯π inter­actions, thereby forming layers lying parallel to (010). Hirshfeld surface analysis was employed to further qu­antify the inter­atomic inter­actions in the crystals of both compounds.

## Abstract 2

The title 4-amino­anti­pyrine Schiff base compounds both deviate from planarity with the phenyl ring and the substituted benzyl­idene ring being inclined to the pyrazole ring mean plane by 54.87 (7) and 22.92 (7)°, respectively, in the first compound and by 60.44 (8) and 12.70 (9)° in the second.

## 1. Chemical context

Anti­pyrine (also known as phenazone) derivatives display anti­oxidant (Bashkatova *et al.*, 2005 ▸), anti-putrefactive (Abd El Rehim *et al.*, 2001 ▸) and optical (Collado *et al.*, 2000 ▸) properties. Among pyrazole analogues, 4-amino-1,5-dimethyl-2-phenyl­pyrazole-3-one, known as 4-amino­anti­pyrine, possesses a free amino group. It has received attention because it exhibits various biological activities, such as anti­fungal, anti­bacterial, anti­malarial, anti­viral, anti-inflammatory and anti­pyretic properties (Nibila *et al.*, 2020 ▸). 4-Amino­anti­pyrine derivatives are also considered to be model compounds in the biological and medical fields (Senthilkumar *et al.*, 2016 ▸). Schiff bases of 4-amino­anti­pyrine and their complexes have a wide range of applications in medicinal, analytical and pharmacological areas (Oudar, 1977 ▸; Zyss, 1979 ▸), and they also possess chemotherapeutic properties (Raman *et al.*, 2007 ▸; Alam & Lee, 2016 ▸). As part of our studies in this area, we now report the syntheses and structures of the title compounds, C_22_H_26_N_4_O (**I**) and C_18_H_16_FN_3_O (**II**). 
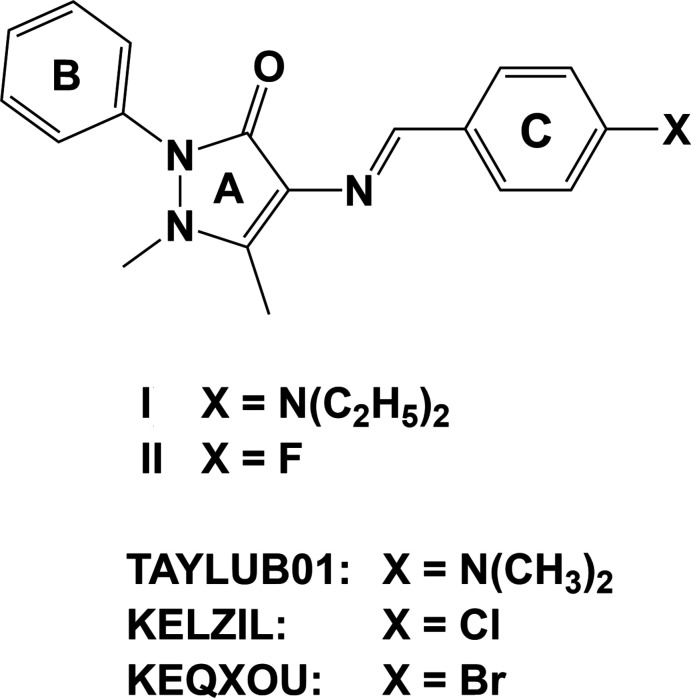


A search of the Cambridge Structural Database (CSD, Version 5.43, last update November 2022; Groom *et al.*, 2016 ▸) gave 31 hits for 4-amino­anti­pyrine structures with a *p*-substituted benzyl­idene ring. Of particular inter­est are the 4-(di­methyl­amino)­benzyl­idene analogue (CSD refcode TAYLUB01; Asiri *et al.*, 2010 ▸) of **I** (both compounds crystallize in the monoclinic space group *C*2/*c*) and the 4-(chloro­amino)­benzyl­idene (KELZIL; Sun *et al.*, 2006 ▸) and 4-(bromo­amino)­benzyl­idene (KEQXOU; Yan *et al.*, 2006 ▸) analogues of **II** (all three compounds crystallize in the ortho­rhom­bic space group *Pbca*). Their mol­ecular structures and Hirshfeld surface analyses are compared to those of the title compounds.

## 2. Structural commentary

**Figure 1:**
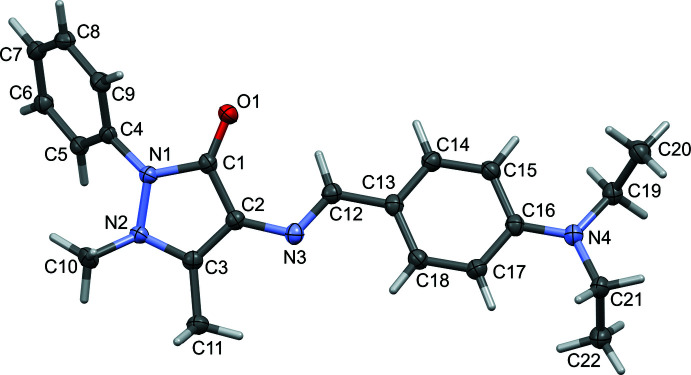
A view of the mol­ecular structure of **I**, with atom labelling. The displacement ellipsoids are drawn at the 50% probability level.

**Figure 3:**
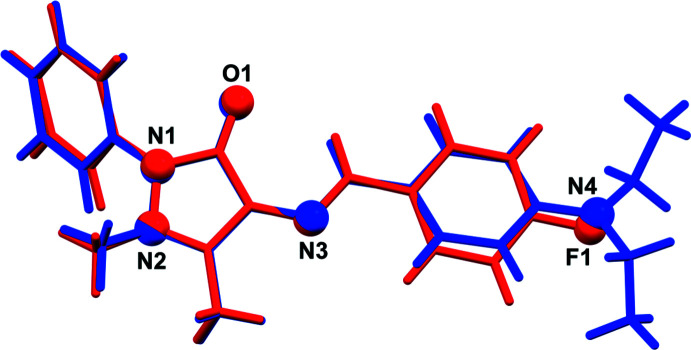
A view of the structural overlap of compounds **I** (blue) and **II** (red); r.m.s. deviation 0.044 Å (*Mercury*; Macrae *et al.*, 2020 ▸). The O, N and F atoms are shown as balls.

The mol­ecular structures of **I** and **II** are illustrated in Figs. 1 ▸ and 2 ▸, respectively. Selected geometric parameters for **I** and **II** and their analogues are given in Table 1 ▸. The various dihedral angles in the five compounds are given in Table 2 ▸. The configuration about the N3=C12 bond is *E*, which favours the presence of an intra­molecular C12—H12⋯O1 hydrogen bond in both compounds (Tables 3 ▸ and 4 ▸, respectively), and in their analogues. The N3=C12 bond length is 1.291 (2) Å in **I** and 1.289 (2) Å in **II**. The pyrazole ring mean plane (*A* = N1/N2/C1–C3; r.m.s. deviations are 0.055 and 0.057 Å for **I** and **II**, respectively) is twisted on the N1—N2 bond in both compounds. The phenyl ring (*B* = C4–C9) and the substituted benzyl­idene ring (*C* = C13–C18) are inclined to the pyrazole ring mean plane *A* by 54.87 (7) and 22.92 (7)°, respectively, in **I** and by 60.44 (8) and 12.70 (9)°, respectively, in **II**. The latter two rings, *B* and *C*, are inclined to each other by 73.98 (6) in **I** and by 71.28 (8)° in **II**. The difference in the conformation of the two structures is illustrated in Fig. 3 ▸ showing the structural overlap (*Mercury*; Macrae *et al.*, 2020 ▸) of mol­ecules **I** and **II**. It can be seen from Table 2 ▸ that the conformation of **I** is similar to that of the 4-(di­methyl­amino)­benzyl­idene analogue (TAYLUB01). However, this is not the case for compound **II**: while the conformation of the 4-(chloro­amino)­benzyl­idene (KELZIL) and 4-(bromo­amino)­benzyl­idene (KEQXOU) analogues of **II** are similar there is a significant difference compared to the conformation of compound **II**. For example, the *A* to *B* dihedral angle is 60.44 (8)° in **II** but is 51.6 (1) and 50.8 (2)°, in the respective analogues. The other dihedral angles are also significantly different, as seen in Table 2 ▸.

The N1 and N2 nitro­gen atoms of the pyrazole ring have pyramidal geometries (see Table 1 ▸), with the sum of their bond angles being 350.5 (1) and 341.5 (1)°, respectively, in **I**, and 348.2 (1) and 351.0 (1)°, respectively, in **II**. The same pyramidal geometries of atoms N1 and N2 are also observed for the various analogues (Table 1 ▸). The bond angles involving atoms N1 and N2 follow the same pattern.

## 3. Supra­molecular features

**Figure 4:**
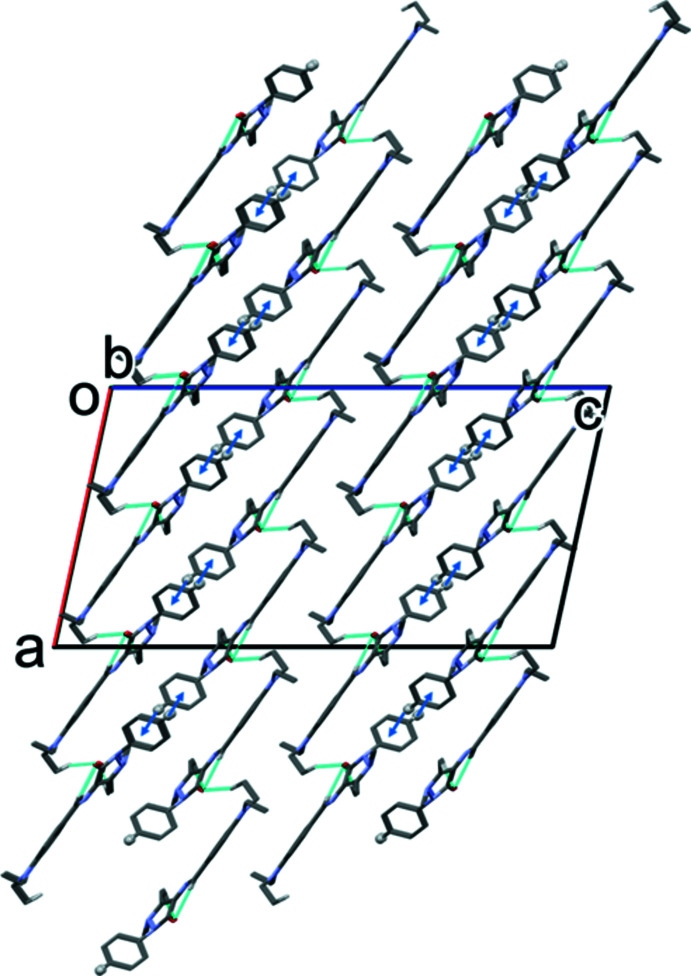
A view along the *b* axis of the crystal packing of **I**. The C—H⋯O hydrogen bonds are shown as dashed lines and the C—H⋯π inter­actions as blue arrows (see Table 3 ▸). Only the H atoms involved in these inter­actions have been included.

In the crystal of **I**, the mol­ecules are linked by C—H⋯O hydrogen bonds, forming slabs lying parallel to the *ab* plane. The slabs are consolidated by C—H⋯π inter­actions (Table 3 ▸ and Fig. 4 ▸).

**Figure 5:**
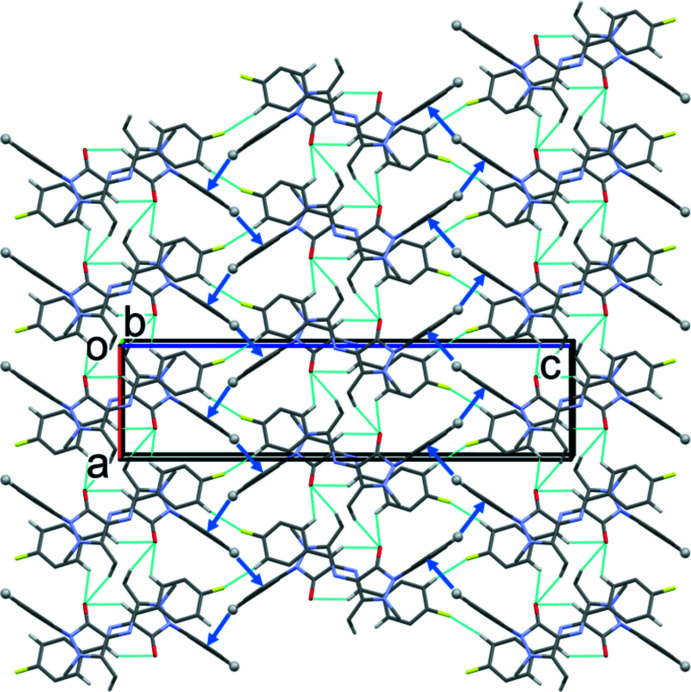
A view along the *b* axis of the crystal packing of **II**. The The C—H⋯O and C—H⋯F hydrogen bonds are shown as dashed lines and the C—H⋯π inter­actions as blue arrows (see Table 4 ▸). Only the H atoms involved in these inter­actions have been included.

In the crystal of **II**, the mol­ecules are linked by C—H⋯O and C—H⋯F hydrogen bonds forming undulating slabs lying parallel to the *ac* plane. Here too, the slabs are strengthened by C—H⋯π inter­actions (Table 4 ▸ and Fig. 5 ▸).

## 4. Hirshfeld surface analysis and two-dimensional fingerprint plots

**Figure 6:**
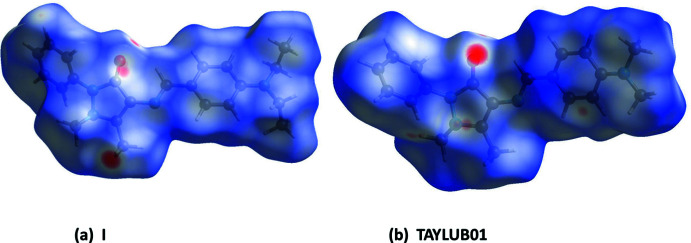
The Hirshfeld surfaces of compounds, (*a*) **I** and (*b*) TAYLUB01 mapped over *d*_norm_ in the colour ranges −0.2834 to 1.4293 and −0.2505 to 1.2511 au., respectively.

**Figure 7:**
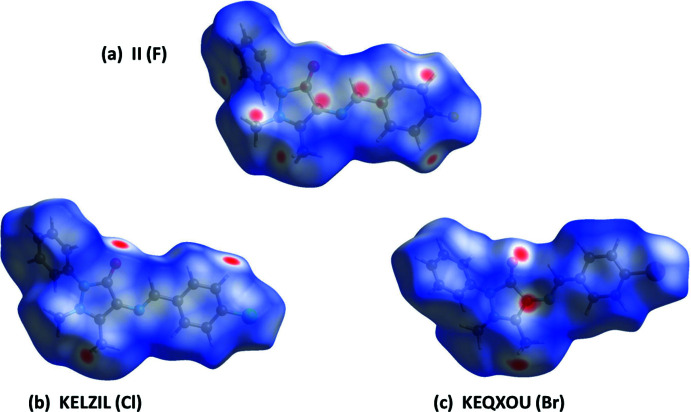
The Hirshfeld surfaces of compounds, (*a*) **II**, (*b*) KELZIL and (*c*) KEQXOU, mapped over *d*_norm_ in the colour ranges −0.2048 to 1.21, −0.2236 to 1.3135 and −0.2367 to 1.3139 au., respectively.

**Figure 8:**
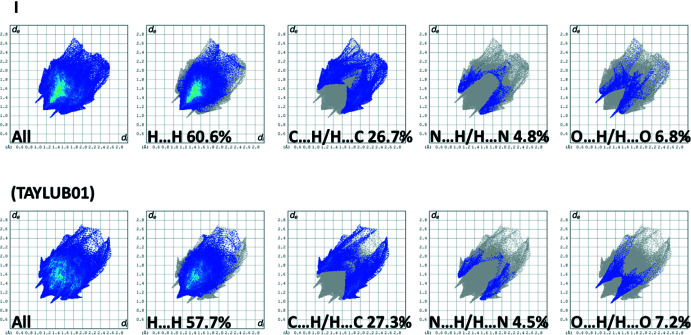
The full two-dimensional fingerprint plots for compounds, (*a*) **I** and (*b*) TAYLUB01, and those delineated into H⋯H, C⋯H/H⋯C, N⋯H/H⋯N and O⋯H/H⋯O contacts.

The Hirshfeld surface analyses and the associated two-dimensional fingerprint plots were performed with *CrystalExplorer17* (Spackman *et al.*, 2021 ▸) following the protocol of Tan *et al.* (2019 ▸). The Hirshfeld surfaces (HS) of **I** and TAYLUB01 are compared in Fig. 6 ▸, and those for **II** and KELZIL and KEQXOU are compared in Fig. 7 ▸. The large red spots indicate that short contacts are significant in the crystal packing of all five crystal structures. The full two-dimensional fingerprint plots for **I** and TAYLUB01, and for **II** and KELZIL and KEQXOU are given in Figs. 8 ▸ and 9 ▸, respectively.

The contributions of the various inter-atomic contacts to the Hirshfeld surfaces for all five compounds are given in Table 5 ▸. In **I** and TAYLUB01 the H⋯H contacts have a major contribution (60.6 and 57.7%, respectively) as do the C⋯H/H⋯C contributions (26.7 and 27.3%, respectively). These are followed by the O⋯H/H⋯O and N⋯H/H⋯N contributions (Table 5 ▸). Other inter-atomic contacts, such as C⋯C and C⋯N/N⋯C contribute less than 2%. For **II**, KELZIL and KEQXOU the H⋯H contacts contribute *ca* 43% for all three compounds, notably less than in **I** and TAYLUB01. The contributions of the C⋯H/H⋯C, N⋯H/H⋯N and O⋯H/H⋯O contacts are similar to those for compound **I** (Table 5 ▸). The halogen⋯H/H⋯halogen contributions vary from 10.5% in **II** to 13.5% in KEQXOU. The C⋯C contributions are 2.7, 2.0 and 2.2%, respectively, while the N⋯C/C⋯N contributions are 1.7, 2.0 and 2.2%, respectively. Both are more significant than for compound **I** and its analogue. The O⋯C/C⋯O contacts contribute less that 1%.

## 5. Energy frameworks

**Figure 10:**
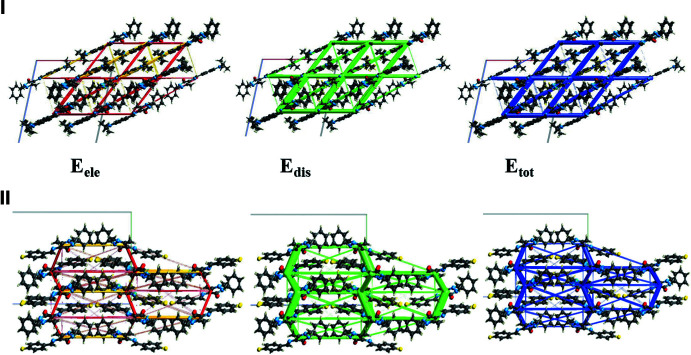
The energy frameworks calculated for **I** viewed along the *b-*axis direction and **II** viewed along the *a-*axis direction showing the electrostatic potential forces (*E*_ele_), the dispersion forces (*E*_dis_) and the total energy diagrams (*E*_tot_).

A comparison of the energy frameworks calculated for **I** and **II**, showing the electrostatic potential forces (*E*_ele_), the dispersion forces (*E*_dis_) and the total energy diagrams (*E*_tot_), are shown in Fig. 10 ▸. The energies were obtained by using wave functions at the HF/3-2IG level of theory. The cylindrical radii are proportional to the relative strength of the corresponding energies (Spackman *et al.*, 2021 ▸; Tan *et al.*, 2019 ▸). They have been adjusted to the same scale factor of 90 with a cut-off value of 6 kJ mol^−1^ within a radius of 6 Å of a central reference mol­ecule. It can be seen that for all five compounds the major contribution to the inter­molecular inter­actions is from dispersion (*E*_dis_), reflecting the absence of classical hydrogen bonds in the crystals. The colour-coded inter­action mappings within a radius of 6 Å of a central reference mol­ecule and the various contributions to the total energy (*E*_tot_) for compounds **I** and **II** are given in Figs. S1 and S2 of the supporting information.

## 6. Database survey

A search of the CSD (CSD, Version 5.43, last update November 2022; Groom *et al.*, 2016 ▸) for benyzyl­idene-substituted 4-amino­anti­pyrine organic structures with R ≤ 0.05, no disorder, no ions, single-crystal analyses only gave more than 90 hits. In all compounds the configuration about the C=N bond is *E*. Various geometrical parameters of these compounds where analysed using *Mercury* (Macrae *et al.*, 2020 ▸). For example, the C=N bond lengths vary from 1.256 to 1.297 Å with a mean value of 1.281 Å (mean s.u. 0.008 Å). For compounds **I** and **II** and their analogues this bond length varies from 1.276 (2) Å for KELZIL (Sun *et al.*, 2006 ▸) to 1.291 (2) Å for **I** (see Table 1 ▸), well within these limits. The C—N—N bond angles within the pyrazole ring vary from *ca* 107.7 to 110.7° with a mean value of 109.3° (mean s.u. 0.5°). The same angles in the title compounds (*i.e*. C1—N1—N2 and C3—N2—N1) and their analogues vary from 106.9 (3)° in KEQXOU (Yan *et al.*, 2006 ▸) to 109.58 (9)° in **I** for the former and 106.23 (9) in **I** to 107.7 (3)° in KEQXOU for the latter. The nitro­gen atoms of the pyrazole ring have pyramidal geometries in all structures.

## 7. Synthesis, crystallization and spectroscopic analyses

Di­ethyl­amino­benzaldehyde (9.08 mmol, 1.744 g) and 1,5-dimethyl-2-phenyl-1*H*-pyrazol-3(2*H*)-one (9.08 mmol, 2.00 g) were added to 100 ml of methanol and the mixture was refluxed at 353 K for a period of 8 h. The solvent was then allowed to evaporate slowly at room temperature. Pale-yellow crystals of compound **I** were obtained after a period of three weeks. Melting point 492 K.

4-Fluoro­benzaldehyde (9.80 mmol, 1.221 g) and 1,5-dimethyl-2-phenyl-1*H*-pyrazol-3(2*H*)-one (9.80 mol, 2.00 g) were added to 100 ml of methanol and the mixture was refluxed at 353 K for a period of 8 h. The solvent was then allowed to evaporate slowly at room temperature. Colourless crystals of compound **II** were obtained after a period of three weeks. Melting point 509 K.

The ^1^H NMR spectra of compounds **I** and **II** were recorded using a Bruker AC 400 MHz spectrometer (Fig. S3 in the supporting information). The compounds were dissolved in CDCl_3_ using tetra­methyl­silane as an inter­nal standard and chemical shifts (δ) are stated in ppm. The imine proton resonated as a sharp singlet peak at 9.63 for **I** and at 9.73 for **II**, whereas the aromatic protons appeared as a multiplet at 6.69–7.74 for **I** and at 7.07–7.87 for **II**. The –NCH_3_ protons of the amino­anti­pyrine unit appeared as a singlet at 3.08 for **I** and 3.16 for **II**. The two ethyl [–N(CH_2_—CH_3_)_2_] group protons in the benzyl­idene moiety of compound **I** appeared as a multiplet at 1.09–1.32 and 3.42–3.45. The methyl protons (C—CH_3_) of the amino­anti­pyrine moiety appeared as a singlet at 2.49 for both **I** and **II**.

FT–IR spectra (KBr pellet) were recorded between 400 and 4000 cm^−1^ (Fig. S4 in the supporting information). The characteristic C=N stretching mode is observed at 1578 for **I**, and at 1577 cm^−1^ for **II**, confirming the formation of the Schiff base compounds. The weak band at 3037 (**I**) and 3035 cm^−1^ (**II**), is assigned to the aromatic C—H stretching vibration. The peaks observed at 1290–1010 (**I**) and 1294–1124 cm^−1^ (**II**) are due to the C—H in-plane bending vibration of the aromatic rings. The bands obtained at 753–976 (**I**) and 757–954 cm^−1^ (**II**) are assigned to C—H out-of-plane bending vibrations. The asymmetric and symmetric stretching vibrations of the methyl group in the 4-amino­anti­pyrine moiety are observed respectively in the ranges of 3010–2970 (**I**) and 2940–2900 cm^−1^ (**II**). The strong peaks at 1650 (**I**) and 1644 cm^−1^ (**II**) correspond to the carbonyl stretching vibrations.

## 8. Refinement

Crystal data, data collection and structure refinement details are summarized in Table 6 ▸. The C-bound H atoms were included in calculated positions and treated as riding atoms: C—H = 0.95–1.0 Å with *U*_iso_(H) = 1.5*U*_eq_(C) for methyl H atoms and = 1.2*U*_eq_(C) for other H atoms.

## Supplementary Material

Crystal structure: contains datablock(s) I, II, Global. DOI: 10.1107/S2056989023004085/hb8065sup1.cif

Structure factors: contains datablock(s) I. DOI: 10.1107/S2056989023004085/hb8065Isup2.hkl

Supporting information file. DOI: 10.1107/S2056989023004085/hb8065Isup4.cml

Structure factors: contains datablock(s) II. DOI: 10.1107/S2056989023004085/hb8065IIsup3.hkl

Supporting information file. DOI: 10.1107/S2056989023004085/hb8065IIsup5.cml

Figures S1-S4. DOI: 10.1107/S2056989023004085/hb8065sup6.pdf

CCDC references: 2261783, 2261784

Additional supporting information:  crystallographic information; 3D view; checkCIF report

## Figures and Tables

**Figure 2:**
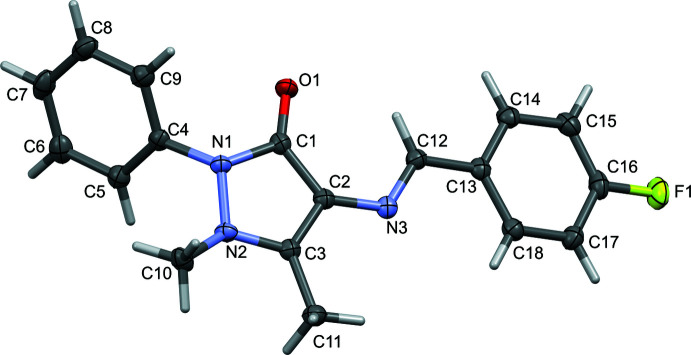
A view of the mol­ecular structure of **II**, with atom labelling. The displacement ellipsoids are drawn at the 50% probability level.

**Figure 9:**
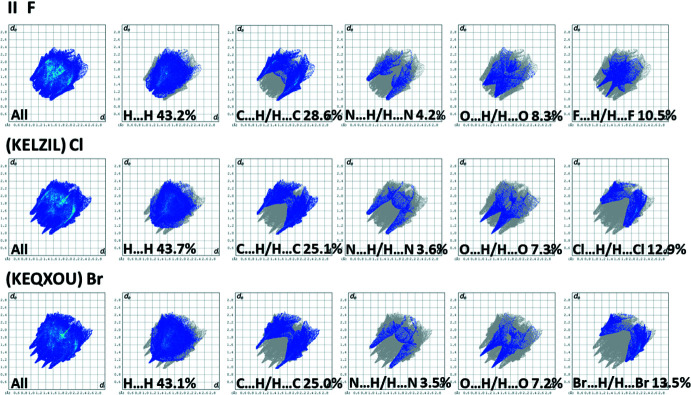
The full two-dimensional fingerprint plots for compounds, (*a*) **II**, (*b*) KELZIL and (*c*) KEQXOU, and those delineated into H⋯H, C⋯H/H⋯C, N⋯H/H⋯N, O⋯H/H⋯O and halogen⋯H/H⋯halogen contacts.

**Table 1:** Selected geometric parameters (Å, °) for **I** and TAYLUB01^*a*^, and for **II** and KELZIL^*b*^ and KEQXOU^*c*^

	**I**	TAYLUB01^*a*^	**II**	KELZIL^*b*^	KEQXOU^*c*^
N3—C12	1.291 (2)	1.288 (2)	1.289 (2)	1.276 (2)	1.279 (5)
C2—N3—C12—C13	–177.11 (11)	173.20 (11)	–175.43 (14)	–176.68 (15)	177.5 (4)
					
C1—N1—N2	109.58 (9)	109.58 (10)	108.75 (12)	108.58 (13)	106.9 (3)
C1—N1—C4	121.78 (10)	122.30 (10)	120.50 (13)	122.40 (13)	122.4 (3)
N2—N1—C4	119.11 (9)	118.13 (10)	118.90 (13)	119.12 (14)	119.8 (3)
Sum	350.47 (9)	350.0 (1)	348.15 (13)	350.10 (13)	349.1 (3)
					
C3—N2—N1	106.23 (9)	106.50 (10)	107.40 (13)	107.34 (13)	107.7 (3)
C3—N2—C10	121.11 (10)	122.30 (11)	125.50 (14)	124.77 (14)	125.1 (3)
N1—N2—C10	114.20 (10)	114.68 (10)	118.09 (13)	117.05 (15)	115.9 (3)
Sum	341.54 (10)	343.48 (10)	350.99 (13)	349.16 (14)	348.7 (3)

Notes: (*a*) Asiri *et al.* (2010 ▸); (*b*) Sun *et al.* 2006 ▸); (*c*) Yan *et al.* 2006 ▸).

**Table 2:** A comparison of various dihedral angles (°) for **I** and TAYLUB01^*a*^, and for **II** and KELZIL^*b*^ and KEQXOU^*c*^ *A* = ring N1/N2/C1–C3, *B* = ring C4–C9, *C* = ring C13–C18 (atom numbering following this paper).

Dihedral angle	**I**	TAYLUB01^*a*^	**II**	KELZIL^*b*^	KEQXOU^*c*^
Planes *A* to *B*	54.87 (7)	55.01 (7)	60.44 (8)	51.6 (1)	50.8 (2)
Planes *A* to *C*	22.92 (7)	19.03 (7)	12.70 (9)	8.7 (1)	9.1 (2)
Planes *B* to *C*	73.98 (6)	73.98 (6)	71.28 (8)	59.0 (1)	59.1 (2)

Notes: (*a*) Asiri *et al.* (2010 ▸); (*b*) Sun *et al.* 2006 ▸); (*c*) Yan *et al.* 2006 ▸).

**Table 3:** Hydrogen-bond geometry (Å, °) for **I** [Chem scheme1] *CgB* is the centroid of ring *B* (C4–C9).

*D*—H⋯*A*	*D*—H	H⋯*A*	*D*⋯*A*	*D*—H⋯*A*
C11—H11*B*⋯O1^i^	0.98	2.33	3.314 (2)	177
C12—H12⋯O1	0.95	2.32	3.028 (2)	131
C22—H22*C*⋯O1^ii^	0.98	2.54	3.466 (2)	157
C7—H7⋯CgB^iii^	0.95	2.79	3.674 (1)	155

Symmetry codes: (i) 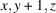 ; (ii) 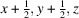 ; (iii) 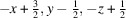 .

**Table 4:** Hydrogen-bond geometry (Å, °) for **II** [Chem scheme1] *CgB* is the centroid of ring *B* (C4–C9).

*D*—H⋯*A*	*D*—H	H⋯*A*	*D*⋯*A*	*D*—H⋯*A*
C11—H11*A*⋯O1^i^	0.98	2.55	3.505 (2)	165
C12—H12*A*⋯O1	0.95	2.36	3.043 (2)	128
C14—H14⋯O1^ii^	0.95	2.57	3.204 (2)	124
C17—H17⋯F1^iii^	0.95	2.50	3.291 (2)	141
C7—H7⋯CgB^iv^	0.95	2.90	3.608 (2)	132

Symmetry codes: (i) 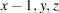 ; (ii) 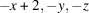 ; (iii) 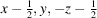 ; (iv) 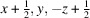 .

**Table 5:** Principal percentage contributions of inter-atomic contacts to the Hirshfeld surfaces of **I**, TAYLUB01^*a*^, **II**, KELZIL^*b*^ and KEQXOU^*c*^

Contact	**I**	TAYLUB01^*a*^	**II**	KELZIL^*b*^	KEQXOU^*c*^
			*X* = F	*X* = Cl	*X* = Br
H⋯H	60.6	57.7	43.2	43.7	43.1
C⋯H/H⋯C	26.7	27.3	28.6	25.1	25.0
N⋯H/H⋯N	4.8	4.5	4.2	3.6	3.5
O⋯H/H⋯O	6.8	7.2	8.3	7.3	7.2
*X*⋯H/H⋯*X*	–	–	10.5	12.9	13.5
C⋯C	0.2	1.3	2.7	3.9	3.9
N⋯C/C⋯N	0.4	1.6	1.7	2.0	2.2
O⋯C/C⋯O	0	0	0.3	0.5	0.5
*X*⋯*X*	–	–	0.4	0.9	1.0

Notes: (*a*) Asiri *et al.* (2010 ▸); (*b*) Sun *et al.* 2006 ▸); (*c*) Yan *et al.* 2006 ▸).

**Table 6:** Experimental details

	**I**	**II**
Crystal data		
Chemical formula	C_22_H_26_N_4_O	C_18_H_16_FN_3_O
*M*_r_	362.47	309.34
Crystal system, space group	Monoclinic, *C*2/*c*	Orthorhombic, *P* *b* *c* *a*
Temperature (K)	120	95
*a*, *b*, *c* (Å)	17.1588 (7), 7.0910 (3), 32.1594 (10)	6.7886 (13), 16.6007 (3), 26.9563 (8)
α, β, γ (°)	90, 102.338 (3), 90	90, 90, 90
*V* (Å^3^)	3822.6 (3)	3037.9 (6)
*Z*	8	8
Radiation type	Cu *K*α	Mo *K*α
μ (mm^−1^)	0.63	0.10
Crystal size (mm)	0.32 × 0.26 × 0.08	0.08 × 0.05 × 0.03

Data collection		
Diffractometer	Xcalibur, Atlas, Gemini ultra	SuperNova, AtlasS2
Absorption correction	Multi-scan (*CrysAlis PRO*; Agilent, 2010 ▸)	Multi-scan (*CrysAlis PRO*; Rigaku OD, 2022 ▸)
*T*_min_, *T*_max_	0.273, 1.000	0.074, 1.000
No. of measured, independent and observed [*I* > 2σ(*I*)] reflections	15635, 3401, 2946	16735, 3041, 2346
*R*_int_	0.034	0.064
(sin θ/λ)_max_ (Å^−1^)	0.598	0.621

Refinement		
*R*[*F* ^2^ > 2σ(*F* ^2^)], *wR*(*F* ^2^), *S*	0.036, 0.093, 1.06	0.042, 0.101, 1.05
No. of reflections	3401	3041
No. of parameters	248	211
H-atom treatment	H-atom parameters constrained	H-atom parameters constrained
Δρ_max_, Δρ_min_ (e Å^−3^)	0.14, −0.21	0.21, −0.21

Computer programs: *CrysAlis PRO* (Agilent, 2010 ▸), *SUPERFLIP* (Palatinus & Chapuis, 2007 ▸; *JANA2006* (Petříček *et al.*, 2014 ▸), *PLATON* (Spek, 2020 ▸); *Mercury* (Macrae *et al.*, 2020 ▸), *SHELXL2018/3* (Sheldrick, 2015 ▸) and *publCIF* (Westrip, 2010 ▸).

## supplementary crystallographic information

### (E)-4-{[4-(Diethylamino)benzylidene]amino}-1,5-dimethyl-2-phenyl-1H-pyrazol-3(2H)-one (I) Crystal data

**Table:**

C_22_H_26_N_4_O	*F*(000) = 1552
*M**_r_* = 362.47	*D*_x_ = 1.260 Mg m^−^^3^
Monoclinic, *C*2/*c*	Cu *K*α radiation, λ = 1.5418 Å
*a* = 17.1588 (7) Å	Cell parameters from 6730 reflections
*b* = 7.0910 (3) Å	θ = 4.2–67.0°
*c* = 32.1594 (10) Å	µ = 0.63 mm^−^^1^
β = 102.338 (3)°	*T* = 120 K
*V* = 3822.6 (3) Å^3^	Plate, yellow
*Z* = 8	0.32 × 0.26 × 0.08 mm

### (E)-4-{[4-(Diethylamino)benzylidene]amino}-1,5-dimethyl-2-phenyl-1H-pyrazol-3(2H)-one (I) Data collection

**Table:**

Xcalibur, Atlas, Gemini ultra diffractometer	3401 independent reflections
Radiation source: Enhance Ultra (Cu) X-ray Source	2946 reflections with *I* > 2σ(*I*)
Mirror monochromator	*R*_int_ = 0.034
Detector resolution: 10.3784 pixels mm^-1^	θ_max_ = 67.1°, θ_min_ = 5.3°
ω scans	*h* = −20→20
Absorption correction: multi-scan (CrysAlisPro; Agilent, 2010)	*k* = −8→8
*T*_min_ = 0.273, *T*_max_ = 1.000	*l* = −32→38
15635 measured reflections	

### (E)-4-{[4-(Diethylamino)benzylidene]amino}-1,5-dimethyl-2-phenyl-1H-pyrazol-3(2H)-one (I) Refinement

**Table:**

Refinement on *F*^2^	Primary atom site location: structure-invariant direct methods
Least-squares matrix: full	Secondary atom site location: difference Fourier map
*R*[*F*^2^ > 2σ(*F*^2^)] = 0.036	Hydrogen site location: inferred from neighbouring sites
*wR*(*F*^2^) = 0.093	H-atom parameters constrained
*S* = 1.06	*w* = 1/[σ^2^(*F*_o_^2^) + (0.0498*P*)^2^ + 1.7059*P*] where *P* = (*F*_o_^2^ + 2*F*_c_^2^)/3
3401 reflections	(Δ/σ)_max_ = 0.001
248 parameters	Δρ_max_ = 0.14 e Å^−^^3^
0 restraints	Δρ_min_ = −0.20 e Å^−^^3^

### (E)-4-{[4-(Diethylamino)benzylidene]amino}-1,5-dimethyl-2-phenyl-1H-pyrazol-3(2H)-one (I) Special details

**Table:**

Geometry. All esds (except the esd in the dihedral angle between two l.s. planes) are estimated using the full covariance matrix. The cell esds are taken into account individually in the estimation of esds in distances, angles and torsion angles; correlations between esds in cell parameters are only used when they are defined by crystal symmetry. An approximate (isotropic) treatment of cell esds is used for estimating esds involving l.s. planes.

### (E)-4-{[4-(Diethylamino)benzylidene]amino}-1,5-dimethyl-2-phenyl-1H-pyrazol-3(2H)-one (I) Fractional atomic coordinates and isotropic or equivalent isotropic displacement parameters (Å^2^)

**Table:**

	*x*	*y*	*z*	*U*_iso_*/*U*_eq_	
O1	0.95050 (6)	0.16596 (12)	0.13672 (3)	0.0258 (2)	
N1	0.91573 (6)	0.42249 (14)	0.17346 (3)	0.0208 (2)	
N2	0.95187 (6)	0.59507 (14)	0.18866 (3)	0.0210 (2)	
N3	1.09105 (6)	0.43910 (15)	0.12703 (3)	0.0223 (2)	
N4	1.37635 (7)	0.11970 (15)	0.04351 (4)	0.0260 (3)	
C1	0.96340 (7)	0.32892 (17)	0.14980 (4)	0.0205 (3)	
C2	1.02614 (7)	0.46216 (17)	0.14650 (4)	0.0206 (3)	
C3	1.01488 (7)	0.61977 (17)	0.16879 (4)	0.0211 (3)	
C4	0.87046 (7)	0.32240 (16)	0.19871 (4)	0.0203 (3)	
C5	0.89105 (7)	0.32887 (17)	0.24292 (4)	0.0223 (3)	
H5	0.933386	0.407554	0.256954	0.027*	
C6	0.84902 (8)	0.21906 (18)	0.26626 (4)	0.0242 (3)	
H6	0.862682	0.222736	0.296476	0.029*	
C7	0.78720 (8)	0.10388 (17)	0.24593 (4)	0.0246 (3)	
H7	0.759273	0.027492	0.262163	0.030*	
C8	0.76628 (8)	0.10055 (18)	0.20181 (4)	0.0256 (3)	
H8	0.723541	0.022873	0.187820	0.031*	
C9	0.80765 (8)	0.21046 (18)	0.17807 (4)	0.0240 (3)	
H9	0.793060	0.209089	0.147860	0.029*	
C10	0.89517 (9)	0.74834 (18)	0.19053 (5)	0.0283 (3)	
H10A	0.859334	0.711170	0.209086	0.042*	
H10B	0.863842	0.773905	0.161857	0.042*	
H10C	0.924493	0.862223	0.201903	0.042*	
C11	1.06192 (8)	0.79734 (18)	0.17469 (4)	0.0265 (3)	
H11A	1.083255	0.818394	0.205111	0.040*	
H11B	1.027446	0.903218	0.162966	0.040*	
H11C	1.106094	0.787545	0.159878	0.040*	
C12	1.10770 (8)	0.27332 (18)	0.11476 (4)	0.0230 (3)	
H12	1.073861	0.170837	0.118039	0.028*	
C13	1.17644 (8)	0.23785 (18)	0.09609 (4)	0.0226 (3)	
C14	1.19818 (8)	0.05234 (19)	0.08887 (4)	0.0253 (3)	
H14	1.166953	−0.048700	0.095862	0.030*	
C15	1.26374 (8)	0.01199 (18)	0.07186 (4)	0.0249 (3)	
H15	1.276951	−0.115738	0.067713	0.030*	
C16	1.31124 (8)	0.15708 (18)	0.06060 (4)	0.0230 (3)	
C17	1.28890 (8)	0.34469 (18)	0.06779 (4)	0.0259 (3)	
H17	1.319282	0.446570	0.060432	0.031*	
C18	1.22408 (8)	0.38183 (18)	0.08523 (4)	0.0242 (3)	
H18	1.211242	0.509177	0.090042	0.029*	
C19	1.40771 (8)	−0.07010 (18)	0.04196 (4)	0.0260 (3)	
H19A	1.395812	−0.144034	0.065967	0.031*	
H19B	1.466432	−0.063114	0.045749	0.031*	
C20	1.37361 (9)	−0.1726 (2)	0.00067 (4)	0.0322 (3)	
H20A	1.396735	−0.299257	0.001640	0.048*	
H20B	1.386511	−0.102288	−0.023225	0.048*	
H20C	1.315550	−0.182293	−0.003084	0.048*	
C21	1.41662 (8)	0.26801 (19)	0.02481 (4)	0.0267 (3)	
H21A	1.376832	0.363552	0.011689	0.032*	
H21B	1.440013	0.212963	0.001953	0.032*	
C22	1.48234 (9)	0.3647 (2)	0.05712 (5)	0.0325 (3)	
H22A	1.509447	0.457307	0.042542	0.049*	
H22B	1.520911	0.270251	0.071042	0.049*	
H22C	1.458950	0.428731	0.078544	0.049*	

### (E)-4-{[4-(Diethylamino)benzylidene]amino}-1,5-dimethyl-2-phenyl-1H-pyrazol-3(2H)-one (I) Atomic displacement parameters (Å^2^)

**Table:**

	*U*^11^	*U*^22^	*U*^33^	*U*^12^	*U*^13^	*U*^23^
O1	0.0313 (5)	0.0211 (4)	0.0271 (5)	−0.0023 (4)	0.0110 (4)	−0.0045 (3)
N1	0.0240 (5)	0.0178 (5)	0.0219 (5)	−0.0019 (4)	0.0078 (4)	−0.0020 (4)
N2	0.0244 (5)	0.0162 (5)	0.0238 (5)	−0.0004 (4)	0.0079 (4)	−0.0014 (4)
N3	0.0229 (5)	0.0243 (5)	0.0202 (5)	0.0009 (4)	0.0058 (4)	0.0007 (4)
N4	0.0271 (6)	0.0242 (6)	0.0295 (6)	0.0001 (4)	0.0123 (5)	−0.0021 (4)
C1	0.0241 (6)	0.0201 (6)	0.0175 (6)	0.0028 (5)	0.0050 (5)	0.0013 (5)
C2	0.0228 (6)	0.0205 (6)	0.0185 (6)	0.0011 (5)	0.0045 (5)	0.0019 (5)
C3	0.0229 (6)	0.0203 (6)	0.0201 (6)	0.0018 (5)	0.0045 (5)	0.0036 (5)
C4	0.0214 (6)	0.0172 (6)	0.0238 (6)	0.0030 (5)	0.0080 (5)	0.0003 (5)
C5	0.0208 (6)	0.0218 (6)	0.0244 (6)	−0.0002 (5)	0.0051 (5)	−0.0028 (5)
C6	0.0273 (7)	0.0241 (6)	0.0224 (6)	0.0014 (5)	0.0082 (5)	−0.0002 (5)
C7	0.0247 (6)	0.0209 (6)	0.0305 (7)	0.0004 (5)	0.0109 (5)	0.0013 (5)
C8	0.0227 (6)	0.0217 (6)	0.0321 (7)	−0.0019 (5)	0.0053 (5)	−0.0031 (5)
C9	0.0250 (6)	0.0239 (6)	0.0225 (6)	0.0002 (5)	0.0035 (5)	−0.0019 (5)
C10	0.0312 (7)	0.0209 (6)	0.0351 (7)	0.0043 (5)	0.0121 (6)	−0.0016 (5)
C11	0.0304 (7)	0.0205 (6)	0.0295 (7)	−0.0026 (5)	0.0084 (5)	0.0001 (5)
C12	0.0249 (6)	0.0242 (6)	0.0201 (6)	−0.0013 (5)	0.0050 (5)	0.0007 (5)
C13	0.0239 (6)	0.0260 (6)	0.0179 (6)	0.0006 (5)	0.0044 (5)	−0.0012 (5)
C14	0.0285 (7)	0.0248 (6)	0.0240 (6)	−0.0032 (5)	0.0083 (5)	−0.0009 (5)
C15	0.0296 (7)	0.0211 (6)	0.0249 (6)	0.0004 (5)	0.0078 (5)	−0.0027 (5)
C16	0.0241 (6)	0.0261 (6)	0.0191 (6)	0.0006 (5)	0.0049 (5)	−0.0014 (5)
C17	0.0277 (7)	0.0234 (6)	0.0284 (7)	−0.0024 (5)	0.0099 (5)	−0.0010 (5)
C18	0.0274 (7)	0.0218 (6)	0.0238 (6)	0.0014 (5)	0.0063 (5)	−0.0016 (5)
C19	0.0258 (7)	0.0272 (7)	0.0261 (7)	0.0032 (5)	0.0078 (5)	−0.0001 (5)
C20	0.0372 (8)	0.0301 (7)	0.0305 (7)	0.0013 (6)	0.0098 (6)	−0.0054 (6)
C21	0.0296 (7)	0.0267 (7)	0.0268 (7)	−0.0008 (5)	0.0126 (5)	−0.0017 (5)
C22	0.0327 (7)	0.0328 (7)	0.0346 (7)	−0.0048 (6)	0.0133 (6)	−0.0063 (6)

### (E)-4-{[4-(Diethylamino)benzylidene]amino}-1,5-dimethyl-2-phenyl-1H-pyrazol-3(2H)-one (I) Geometric parameters (Å, º)

**Table:**

O1—C1	1.2335 (15)	C11—H11A	0.9800
N1—C1	1.3978 (16)	C11—H11B	0.9800
N1—N2	1.4112 (14)	C11—H11C	0.9800
N1—C4	1.4269 (16)	C12—C13	1.4551 (18)
N2—C3	1.3796 (16)	C12—H12	0.9500
N2—C10	1.4685 (16)	C13—C18	1.3982 (18)
N3—C12	1.2913 (17)	C13—C14	1.4002 (18)
N3—C2	1.3991 (16)	C14—C15	1.3817 (19)
N4—C16	1.3720 (17)	C14—H14	0.9500
N4—C19	1.4543 (17)	C15—C16	1.4073 (18)
N4—C21	1.4569 (17)	C15—H15	0.9500
C1—C2	1.4533 (18)	C16—C17	1.4172 (18)
C2—C3	1.3635 (17)	C17—C18	1.3737 (19)
C3—C11	1.4858 (18)	C17—H17	0.9500
C4—C9	1.3881 (18)	C18—H18	0.9500
C4—C5	1.3907 (18)	C19—C20	1.5170 (19)
C5—C6	1.3860 (18)	C19—H19A	0.9900
C5—H5	0.9500	C19—H19B	0.9900
C6—C7	1.3868 (19)	C20—H20A	0.9800
C6—H6	0.9500	C20—H20B	0.9800
C7—C8	1.3875 (19)	C20—H20C	0.9800
C7—H7	0.9500	C21—C22	1.523 (2)
C8—C9	1.3877 (19)	C21—H21A	0.9900
C8—H8	0.9500	C21—H21B	0.9900
C9—H9	0.9500	C22—H22A	0.9800
C10—H10A	0.9800	C22—H22B	0.9800
C10—H10B	0.9800	C22—H22C	0.9800
C10—H10C	0.9800		
			
C1—N1—N2	109.58 (9)	H11A—C11—H11C	109.5
C1—N1—C4	121.78 (10)	H11B—C11—H11C	109.5
N2—N1—C4	119.11 (9)	N3—C12—C13	122.35 (12)
C3—N2—N1	106.23 (9)	N3—C12—H12	118.8
C3—N2—C10	121.11 (10)	C13—C12—H12	118.8
N1—N2—C10	114.20 (10)	C18—C13—C14	116.99 (11)
C12—N3—C2	119.50 (11)	C18—C13—C12	123.07 (11)
C16—N4—C19	122.10 (11)	C14—C13—C12	119.93 (11)
C16—N4—C21	121.63 (11)	C15—C14—C13	121.91 (12)
C19—N4—C21	116.24 (10)	C15—C14—H14	119.0
O1—C1—N1	123.17 (11)	C13—C14—H14	119.0
O1—C1—C2	131.75 (11)	C14—C15—C16	121.06 (12)
N1—C1—C2	105.07 (10)	C14—C15—H15	119.5
C3—C2—N3	123.15 (11)	C16—C15—H15	119.5
C3—C2—C1	107.70 (11)	N4—C16—C15	121.87 (11)
N3—C2—C1	129.03 (11)	N4—C16—C17	121.24 (12)
C2—C3—N2	110.64 (11)	C15—C16—C17	116.90 (12)
C2—C3—C11	128.87 (12)	C18—C17—C16	121.17 (12)
N2—C3—C11	120.44 (11)	C18—C17—H17	119.4
C9—C4—C5	120.63 (11)	C16—C17—H17	119.4
C9—C4—N1	118.27 (11)	C17—C18—C13	121.98 (12)
C5—C4—N1	121.01 (11)	C17—C18—H18	119.0
C6—C5—C4	119.15 (12)	C13—C18—H18	119.0
C6—C5—H5	120.4	N4—C19—C20	113.39 (11)
C4—C5—H5	120.4	N4—C19—H19A	108.9
C5—C6—C7	120.64 (12)	C20—C19—H19A	108.9
C5—C6—H6	119.7	N4—C19—H19B	108.9
C7—C6—H6	119.7	C20—C19—H19B	108.9
C6—C7—C8	119.79 (12)	H19A—C19—H19B	107.7
C6—C7—H7	120.1	C19—C20—H20A	109.5
C8—C7—H7	120.1	C19—C20—H20B	109.5
C7—C8—C9	120.14 (12)	H20A—C20—H20B	109.5
C7—C8—H8	119.9	C19—C20—H20C	109.5
C9—C8—H8	119.9	H20A—C20—H20C	109.5
C8—C9—C4	119.62 (12)	H20B—C20—H20C	109.5
C8—C9—H9	120.2	N4—C21—C22	113.00 (11)
C4—C9—H9	120.2	N4—C21—H21A	109.0
N2—C10—H10A	109.5	C22—C21—H21A	109.0
N2—C10—H10B	109.5	N4—C21—H21B	109.0
H10A—C10—H10B	109.5	C22—C21—H21B	109.0
N2—C10—H10C	109.5	H21A—C21—H21B	107.8
H10A—C10—H10C	109.5	C21—C22—H22A	109.5
H10B—C10—H10C	109.5	C21—C22—H22B	109.5
C3—C11—H11A	109.5	H22A—C22—H22B	109.5
C3—C11—H11B	109.5	C21—C22—H22C	109.5
H11A—C11—H11B	109.5	H22A—C22—H22C	109.5
C3—C11—H11C	109.5	H22B—C22—H22C	109.5
			
C1—N1—N2—C3	9.12 (13)	C4—C5—C6—C7	0.04 (19)
C4—N1—N2—C3	156.03 (10)	C5—C6—C7—C8	−1.04 (19)
C1—N1—N2—C10	145.25 (10)	C6—C7—C8—C9	0.74 (19)
C4—N1—N2—C10	−67.83 (14)	C7—C8—C9—C4	0.54 (19)
N2—N1—C1—O1	171.84 (11)	C5—C4—C9—C8	−1.55 (18)
C4—N1—C1—O1	25.96 (18)	N1—C4—C9—C8	175.24 (11)
N2—N1—C1—C2	−6.89 (13)	C2—N3—C12—C13	−177.11 (11)
C4—N1—C1—C2	−152.77 (11)	N3—C12—C13—C18	−7.96 (19)
C12—N3—C2—C3	164.73 (12)	N3—C12—C13—C14	171.03 (12)
C12—N3—C2—C1	−10.89 (19)	C18—C13—C14—C15	0.13 (19)
O1—C1—C2—C3	−176.49 (13)	C12—C13—C14—C15	−178.92 (12)
N1—C1—C2—C3	2.09 (13)	C13—C14—C15—C16	−0.7 (2)
O1—C1—C2—N3	−0.3 (2)	C19—N4—C16—C15	−9.62 (19)
N1—C1—C2—N3	178.24 (11)	C21—N4—C16—C15	168.66 (12)
N3—C2—C3—N2	−172.83 (11)	C19—N4—C16—C17	170.24 (12)
C1—C2—C3—N2	3.60 (14)	C21—N4—C16—C17	−11.49 (19)
N3—C2—C3—C11	4.6 (2)	C14—C15—C16—N4	−179.73 (12)
C1—C2—C3—C11	−178.95 (12)	C14—C15—C16—C17	0.41 (18)
N1—N2—C3—C2	−7.78 (13)	N4—C16—C17—C18	−179.46 (12)
C10—N2—C3—C2	−140.20 (12)	C15—C16—C17—C18	0.40 (18)
N1—N2—C3—C11	174.52 (11)	C16—C17—C18—C13	−1.0 (2)
C10—N2—C3—C11	42.11 (17)	C14—C13—C18—C17	0.71 (19)
C1—N1—C4—C9	−66.68 (16)	C12—C13—C18—C17	179.72 (12)
N2—N1—C4—C9	150.54 (11)	C16—N4—C19—C20	92.75 (14)
C1—N1—C4—C5	110.10 (13)	C21—N4—C19—C20	−85.62 (14)
N2—N1—C4—C5	−32.67 (16)	C16—N4—C21—C22	88.73 (15)
C9—C4—C5—C6	1.26 (18)	C19—N4—C21—C22	−92.90 (14)
N1—C4—C5—C6	−175.45 (11)		

### (E)-4-{[4-(Diethylamino)benzylidene]amino}-1,5-dimethyl-2-phenyl-1H-pyrazol-3(2H)-one (I) Hydrogen-bond geometry (Å, º)

*CgB* is the centroid of ring *B* (C4–C9).

**Table:**

*D*—H···*A*	*D*—H	H···*A*	*D*···*A*	*D*—H···*A*
C11—H11*B*···O1^i^	0.98	2.33	3.314 (2)	177
C12—H12···O1	0.95	2.32	3.028 (2)	131
C22—H22*C*···O1^ii^	0.98	2.54	3.466 (2)	157
C7—H7···CgB^iii^	0.95	2.79	3.674 (1)	155

Symmetry codes: (i) *x*, *y*+1, *z*; (ii) *x*+1/2, *y*+1/2, *z*; (iii) −*x*+3/2, *y*−1/2, −*z*+1/2.

### (E)-4-[(4-Fluorobenzylidene)amino]-1,5-dimethyl-2-phenyl-1H-pyrazol-3(2H)-one (II) Crystal data

**Table:**

C_18_H_16_FN_3_O	*D*_x_ = 1.353 Mg m^−^^3^
*M**_r_* = 309.34	Mo *K*α radiation, λ = 0.71073 Å
Orthorhombic, *P**b**c**a*	Cell parameters from 3075 reflections
*a* = 6.7886 (13) Å	θ = 5.3–73.0°
*b* = 16.6007 (3) Å	µ = 0.10 mm^−^^1^
*c* = 26.9563 (8) Å	*T* = 95 K
*V* = 3037.9 (6) Å^3^	Block, colourless
*Z* = 8	0.08 × 0.05 × 0.03 mm
*F*(000) = 1296	

### (E)-4-[(4-Fluorobenzylidene)amino]-1,5-dimethyl-2-phenyl-1H-pyrazol-3(2H)-one (II) Data collection

**Table:**

SuperNova, AtlasS2 diffractometer	3041 independent reflections
Radiation source: X-ray tube	2346 reflections with *I* > 2σ(*I*)
Mirror monochromator	*R*_int_ = 0.064
Detector resolution: 5.2027 pixels mm^-1^	θ_max_ = 26.2°, θ_min_ = 1.5°
ω scans	*h* = −7→8
Absorption correction: multi-scan (CrysAlisPro; Rigaku OD, 2022)	*k* = −20→20
*T*_min_ = 0.074, *T*_max_ = 1.000	*l* = −21→33
16735 measured reflections	

### (E)-4-[(4-Fluorobenzylidene)amino]-1,5-dimethyl-2-phenyl-1H-pyrazol-3(2H)-one (II) Refinement

**Table:**

Refinement on *F*^2^	Secondary atom site location: difference Fourier map
Least-squares matrix: full	Hydrogen site location: inferred from neighbouring sites
*R*[*F*^2^ > 2σ(*F*^2^)] = 0.042	H-atom parameters constrained
*wR*(*F*^2^) = 0.101	*w* = 1/[σ^2^(*F*_o_^2^) + (0.0413*P*)^2^ + 0.3986*P*] where *P* = (*F*_o_^2^ + 2*F*_c_^2^)/3
*S* = 1.05	(Δ/σ)_max_ < 0.001
3041 reflections	Δρ_max_ = 0.21 e Å^−^^3^
211 parameters	Δρ_min_ = −0.21 e Å^−^^3^
0 restraints	Extinction correction: (*SHELXL2018/3*; Sheldrick, 2015), Fc^*^=kFc[1+0.001xFc^2^λ^3^/sin(2θ)]^-1/4^
Primary atom site location: structure-invariant direct methods	Extinction coefficient: 0.0025 (5)

### (E)-4-[(4-Fluorobenzylidene)amino]-1,5-dimethyl-2-phenyl-1H-pyrazol-3(2H)-one (II) Special details

**Table:**

Geometry. All esds (except the esd in the dihedral angle between two l.s. planes) are estimated using the full covariance matrix. The cell esds are taken into account individually in the estimation of esds in distances, angles and torsion angles; correlations between esds in cell parameters are only used when they are defined by crystal symmetry. An approximate (isotropic) treatment of cell esds is used for estimating esds involving l.s. planes.

### (E)-4-[(4-Fluorobenzylidene)amino]-1,5-dimethyl-2-phenyl-1H-pyrazol-3(2H)-one (II) Fractional atomic coordinates and isotropic or equivalent isotropic displacement parameters (Å^2^)

**Table:**

	*x*	*y*	*z*	*U*_iso_*/*U*_eq_	
F1	0.87181 (18)	0.01263 (7)	−0.22907 (4)	0.0355 (3)	
O1	0.72869 (16)	0.08876 (7)	0.07747 (4)	0.0227 (3)	
N1	0.45169 (19)	0.15388 (8)	0.10880 (5)	0.0191 (3)	
N2	0.28459 (19)	0.19055 (8)	0.08765 (5)	0.0199 (3)	
N3	0.48528 (19)	0.10471 (8)	−0.02205 (5)	0.0188 (3)	
C1	0.5616 (2)	0.11647 (10)	0.07073 (6)	0.0185 (3)	
C2	0.4406 (2)	0.12362 (9)	0.02704 (6)	0.0182 (3)	
C3	0.2737 (2)	0.16588 (9)	0.03975 (6)	0.0184 (3)	
C4	0.5493 (2)	0.19326 (10)	0.14907 (6)	0.0196 (3)	
C5	0.5650 (2)	0.27676 (11)	0.15110 (6)	0.0226 (4)	
H5	0.504845	0.309414	0.126438	0.027*	
C6	0.6700 (2)	0.31165 (12)	0.18982 (7)	0.0274 (4)	
H6	0.680539	0.368625	0.191832	0.033*	
C7	0.7597 (2)	0.26390 (13)	0.22553 (7)	0.0313 (4)	
H7	0.832965	0.288135	0.251573	0.038*	
C8	0.7421 (3)	0.18061 (13)	0.22317 (7)	0.0300 (4)	
H8	0.802971	0.147933	0.247699	0.036*	
C9	0.6357 (2)	0.14503 (11)	0.18500 (6)	0.0242 (4)	
H9	0.622161	0.088118	0.183518	0.029*	
C10	0.1160 (2)	0.20736 (11)	0.11996 (6)	0.0240 (4)	
H10A	0.161622	0.234823	0.150023	0.036*	
H10B	0.051684	0.156623	0.129077	0.036*	
H10C	0.021908	0.241881	0.102403	0.036*	
C11	0.0992 (2)	0.18413 (11)	0.00855 (7)	0.0252 (4)	
H11C	0.124258	0.166697	−0.025605	0.038*	
H11B	0.073898	0.242251	0.009018	0.038*	
H11A	−0.015913	0.155503	0.021628	0.038*	
C12	0.6468 (2)	0.06772 (10)	−0.03275 (6)	0.0196 (3)	
H12A	0.730837	0.049874	−0.006810	0.024*	
C13	0.7020 (2)	0.05290 (9)	−0.08456 (6)	0.0196 (3)	
C14	0.8853 (3)	0.01856 (10)	−0.09495 (6)	0.0231 (4)	
H14	0.970961	0.004617	−0.068426	0.028*	
C15	0.9441 (3)	0.00454 (11)	−0.14364 (6)	0.0257 (4)	
H15	1.068284	−0.019112	−0.150837	0.031*	
C16	0.8166 (3)	0.02601 (11)	−0.18100 (6)	0.0265 (4)	
C17	0.6341 (3)	0.06039 (11)	−0.17255 (6)	0.0248 (4)	
H17	0.550125	0.074561	−0.199357	0.030*	
C18	0.5772 (2)	0.07358 (10)	−0.12379 (6)	0.0222 (3)	
H18	0.452267	0.096883	−0.117004	0.027*	

### (E)-4-[(4-Fluorobenzylidene)amino]-1,5-dimethyl-2-phenyl-1H-pyrazol-3(2H)-one (II) Atomic displacement parameters (Å^2^)

**Table:**

	*U*^11^	*U*^22^	*U*^33^	*U*^12^	*U*^13^	*U*^23^
F1	0.0454 (6)	0.0410 (7)	0.0201 (5)	0.0028 (5)	0.0106 (5)	−0.0031 (4)
O1	0.0174 (5)	0.0287 (6)	0.0220 (6)	0.0060 (5)	−0.0013 (5)	−0.0022 (5)
N1	0.0162 (6)	0.0213 (7)	0.0197 (6)	0.0024 (5)	−0.0019 (5)	−0.0016 (5)
N2	0.0145 (6)	0.0222 (7)	0.0231 (7)	0.0029 (5)	−0.0011 (5)	−0.0002 (5)
N3	0.0198 (6)	0.0180 (7)	0.0187 (6)	−0.0020 (5)	0.0014 (5)	0.0002 (5)
C1	0.0189 (7)	0.0171 (8)	0.0196 (8)	−0.0015 (6)	0.0023 (6)	0.0002 (6)
C2	0.0187 (7)	0.0154 (7)	0.0206 (8)	−0.0021 (6)	0.0001 (6)	0.0019 (6)
C3	0.0181 (7)	0.0161 (7)	0.0211 (8)	−0.0032 (6)	−0.0003 (6)	0.0011 (6)
C4	0.0135 (7)	0.0261 (9)	0.0192 (8)	0.0000 (6)	0.0020 (6)	−0.0015 (6)
C5	0.0187 (7)	0.0266 (9)	0.0223 (8)	−0.0009 (7)	0.0020 (6)	−0.0011 (7)
C6	0.0201 (8)	0.0320 (10)	0.0302 (9)	−0.0052 (7)	0.0045 (7)	−0.0079 (8)
C7	0.0190 (8)	0.0497 (12)	0.0251 (9)	−0.0032 (8)	−0.0009 (7)	−0.0106 (8)
C8	0.0217 (8)	0.0458 (12)	0.0227 (8)	0.0046 (8)	−0.0025 (7)	0.0005 (8)
C9	0.0187 (7)	0.0310 (10)	0.0229 (8)	0.0023 (7)	0.0027 (7)	0.0008 (7)
C10	0.0180 (7)	0.0254 (9)	0.0287 (9)	0.0011 (6)	0.0039 (7)	−0.0043 (7)
C11	0.0220 (8)	0.0251 (9)	0.0284 (9)	0.0035 (7)	−0.0046 (7)	−0.0002 (7)
C12	0.0205 (7)	0.0175 (8)	0.0208 (8)	−0.0013 (6)	−0.0019 (6)	0.0006 (6)
C13	0.0227 (8)	0.0151 (8)	0.0210 (8)	−0.0023 (6)	0.0011 (7)	0.0005 (6)
C14	0.0256 (8)	0.0197 (8)	0.0240 (8)	0.0012 (7)	0.0004 (7)	0.0020 (6)
C15	0.0269 (8)	0.0218 (8)	0.0284 (9)	0.0030 (7)	0.0062 (7)	−0.0005 (7)
C16	0.0366 (9)	0.0229 (9)	0.0199 (8)	−0.0027 (7)	0.0083 (7)	−0.0028 (6)
C17	0.0285 (8)	0.0257 (9)	0.0202 (8)	−0.0013 (7)	−0.0014 (7)	0.0002 (7)
C18	0.0227 (8)	0.0201 (8)	0.0238 (8)	0.0001 (6)	0.0015 (7)	−0.0001 (6)

### (E)-4-[(4-Fluorobenzylidene)amino]-1,5-dimethyl-2-phenyl-1H-pyrazol-3(2H)-one (II) Geometric parameters (Å, º)

**Table:**

F1—C16	1.367 (2)	C8—H8	0.9500
O1—C1	1.237 (2)	C9—H9	0.9500
N1—N2	1.4080 (18)	C10—H10A	0.9800
N1—C1	1.413 (2)	C10—H10B	0.9800
N1—C4	1.430 (2)	C10—H10C	0.9800
N2—C3	1.356 (2)	C11—H11C	0.9800
N2—C10	1.465 (2)	C11—H11B	0.9800
N3—C12	1.289 (2)	C11—H11A	0.9800
N3—C2	1.394 (2)	C12—C13	1.467 (2)
C1—C2	1.441 (2)	C12—H12A	0.9500
C2—C3	1.376 (2)	C13—C14	1.397 (2)
C3—C11	1.484 (2)	C13—C18	1.398 (2)
C4—C9	1.387 (2)	C14—C15	1.392 (2)
C4—C5	1.391 (2)	C14—H14	0.9500
C5—C6	1.390 (2)	C15—C16	1.375 (3)
C5—H5	0.9500	C15—H15	0.9500
C6—C7	1.388 (3)	C16—C17	1.383 (3)
C6—H6	0.9500	C17—C18	1.387 (2)
C7—C8	1.389 (3)	C17—H17	0.9500
C7—H7	0.9500	C18—H18	0.9500
C8—C9	1.389 (3)		
			
N2—N1—C1	108.75 (12)	N2—C10—H10A	109.5
C1—N1—C4	120.50 (13)	N2—C10—H10B	109.5
N2—N1—C4	118.90 (13)	H10A—C10—H10B	109.5
C3—N2—N1	107.40 (13)	N2—C10—H10C	109.5
C3—N2—C10	125.50 (14)	H10A—C10—H10C	109.5
N1—N2—C10	118.09 (13)	H10B—C10—H10C	109.5
C12—N3—C2	120.32 (14)	C3—C11—H11C	109.5
O1—C1—N1	122.75 (15)	C3—C11—H11B	109.5
O1—C1—C2	132.33 (15)	H11C—C11—H11B	109.5
N1—C1—C2	104.85 (13)	C3—C11—H11A	109.5
C3—C2—N3	122.05 (14)	H11C—C11—H11A	109.5
C3—C2—C1	107.94 (14)	H11B—C11—H11A	109.5
N3—C2—C1	129.30 (14)	N3—C12—C13	120.68 (15)
N2—C3—C2	110.24 (14)	N3—C12—H12A	119.7
N2—C3—C11	121.43 (14)	C13—C12—H12A	119.7
C2—C3—C11	128.33 (15)	C14—C13—C18	119.24 (15)
C9—C4—C5	121.03 (16)	C14—C13—C12	119.14 (15)
C9—C4—N1	117.53 (15)	C18—C13—C12	121.62 (15)
C5—C4—N1	121.38 (15)	C15—C14—C13	120.84 (16)
C6—C5—C4	118.93 (16)	C15—C14—H14	119.6
C6—C5—H5	120.5	C13—C14—H14	119.6
C4—C5—H5	120.5	C16—C15—C14	117.85 (16)
C7—C6—C5	120.50 (18)	C16—C15—H15	121.1
C7—C6—H6	119.7	C14—C15—H15	121.1
C5—C6—H6	119.7	F1—C16—C15	118.67 (16)
C6—C7—C8	119.95 (17)	F1—C16—C17	117.94 (16)
C6—C7—H7	120.0	C15—C16—C17	123.39 (16)
C8—C7—H7	120.0	C16—C17—C18	118.07 (16)
C9—C8—C7	120.12 (18)	C16—C17—H17	121.0
C9—C8—H8	119.9	C18—C17—H17	121.0
C7—C8—H8	119.9	C17—C18—C13	120.62 (16)
C4—C9—C8	119.45 (18)	C17—C18—H18	119.7
C4—C9—H9	120.3	C13—C18—H18	119.7
C8—C9—H9	120.3		
			
C1—N1—N2—C3	9.45 (17)	N2—N1—C4—C5	−36.0 (2)
C4—N1—N2—C3	152.54 (14)	C1—N1—C4—C5	102.73 (18)
C1—N1—N2—C10	158.44 (14)	C9—C4—C5—C6	0.3 (2)
C4—N1—N2—C10	−58.47 (19)	N1—C4—C5—C6	−176.80 (14)
N2—N1—C1—O1	170.21 (15)	C4—C5—C6—C7	0.7 (2)
C4—N1—C1—O1	27.8 (2)	C5—C6—C7—C8	−1.0 (3)
N2—N1—C1—C2	−7.02 (17)	C6—C7—C8—C9	0.3 (3)
C4—N1—C1—C2	−149.42 (14)	C5—C4—C9—C8	−1.0 (2)
C12—N3—C2—C3	177.33 (15)	N1—C4—C9—C8	176.19 (14)
C12—N3—C2—C1	8.2 (3)	C7—C8—C9—C4	0.7 (2)
O1—C1—C2—C3	−174.69 (18)	C2—N3—C12—C13	−175.43 (14)
N1—C1—C2—C3	2.15 (17)	N3—C12—C13—C14	174.82 (15)
O1—C1—C2—N3	−4.3 (3)	N3—C12—C13—C18	−4.0 (2)
N1—C1—C2—N3	172.51 (15)	C18—C13—C14—C15	−0.3 (2)
N1—N2—C3—C2	−8.11 (18)	C12—C13—C14—C15	−179.17 (16)
C10—N2—C3—C2	−154.17 (15)	C13—C14—C15—C16	0.4 (3)
N1—N2—C3—C11	171.33 (14)	C14—C15—C16—F1	−179.96 (16)
C10—N2—C3—C11	25.3 (2)	C14—C15—C16—C17	−0.2 (3)
N3—C2—C3—N2	−167.49 (14)	F1—C16—C17—C18	179.62 (15)
C1—C2—C3—N2	3.71 (18)	C15—C16—C17—C18	−0.2 (3)
N3—C2—C3—C11	13.1 (3)	C16—C17—C18—C13	0.3 (3)
C1—C2—C3—C11	−175.67 (16)	C14—C13—C18—C17	−0.1 (2)
N2—N1—C4—C9	146.82 (14)	C12—C13—C18—C17	178.78 (15)
C1—N1—C4—C9	−74.49 (19)		

### (E)-4-[(4-Fluorobenzylidene)amino]-1,5-dimethyl-2-phenyl-1H-pyrazol-3(2H)-one (II) Hydrogen-bond geometry (Å, º)

*CgB* is the centroid of ring *B* (C4–C9).

**Table:**

*D*—H···*A*	*D*—H	H···*A*	*D*···*A*	*D*—H···*A*
C11—H11*A*···O1^i^	0.98	2.55	3.505 (2)	165
C12—H12*A*···O1	0.95	2.36	3.043 (2)	128
C14—H14···O1^ii^	0.95	2.57	3.204 (2)	124
C17—H17···F1^iii^	0.95	2.50	3.291 (2)	141
C7—H7···CgB^iv^	0.95	2.90	3.608 (2)	132

Symmetry codes: (i) *x*−1, *y*, *z*; (ii) −*x*+2, −*y*, −*z*; (iii) *x*−1/2, *y*, −*z*−1/2; (iv) *x*+1/2, *y*, −*z*+1/2.

## References

[bb1] Abd El Rehim, S. S., Ibrahim, M. A. M. & Khalid, K. F. (2001). *Mater. Chem. Phys.* **70**, 268–273.

[bb2] Agilent (2010). *CrysAlis PRO*. Agilent Technologies, Yarnton, England.

[bb4] Alam, M. S. & Lee, D.-U. (2016). *EXCLI J*. **15**, 614–629. 10.17179/excli2016-477 PMC5225685 28096791

[bb5] Asiri, A. M., Khan, S. A., Tan, K. W. & Ng, S. W. (2010). *Acta Cryst.* E**66**, o1751. 10.1107/S1600536810023536 PMC3006679 21587967

[bb6] Bashkatova, N. V., Korotkova, E. I., Karbainov, Y. A., Yagovkin, A. Y. & Bakibaev, A. A. (2005). *J. Pharm. Biomed. Anal.* **37**, 1143–1147. 10.1016/j.jpba.2004.11.046 15862698

[bb7] Collado, M. S., Mantovani, V. E., Goicoechea, H. C. & Olivieri, A. C. (2000). *Talanta*, **52**, 909–920. 10.1016/s0039-9140(00)00443-4 18968052

[bb9] Groom, C. R., Bruno, I. J., Lightfoot, M. P. & Ward, S. C. (2016). *Acta Cryst.* B**72**, 171–179. 10.1107/S2052520616003954 PMC4822653 27048719

[bb10] Macrae, C. F., Sovago, I., Cottrell, S. J., Galek, P. T. A., McCabe, P., Pidcock, E., Platings, M., Shields, G. P., Stevens, J. S., Towler, M. & Wood, P. A. (2020). *J. Appl. Cryst.* **53**, 226–235. 10.1107/S1600576719014092 PMC6998782 32047413

[bb11] Nibila, T. A., Shameera Ahamed, T. K., Soufeena, P. P., Muraleedharan, K., Peiyat, P. & Aravindakshan, K. K. (2020). *Results Chem.* **2**, 100062. https://doi.org/10.1016/j.rechem.2020.100062

[bb12] Oudar, J. L. (1977). *J. Chem. Phys.* **67**, 446–457.

[bb13] Palatinus, L. & Chapuis, G. (2007). *J. Appl. Cryst.* **40**, 786–790.

[bb14] Petříček, V., Dušek, M. & Palatinus, L. (2014). *Z. Kristallogr.* **229**, 345–352.

[bb15] Raman, N., Dhaveethu Raja, J. & Sakthivel, A. (2007). *J. Chem. Sci.* **119**, 303–310.

[bb16] Rigaku OD (2022). *CrysAlis PRO*. Rigaku Oxford Diffraction Ltd, Yarnton, England.

[bb17] Senthilkumar, K., Thirumoorthy, K., Dragonetti, C., Marinotto, D., Righetto, S., Colombo, A., Haukka, M. & Palanisami, N. (2016). *Dalton Trans.* **45**, 11939–11943. 10.1039/c6dt01590e 27402322

[bb18] Sheldrick, G. M. (2015). *Acta Cryst.* C**71**, 3–8.

[bb19] Spackman, P. R., Turner, M. J., McKinnon, J. J., Wolff, S. K., Grimwood, D. J., Jayatilaka, D. & Spackman, M. A. (2021). *J. Appl. Cryst.* **54**, 1006–1011. 10.1107/S1600576721002910 PMC8202033 34188619

[bb20] Spek, A. L. (2020). *Acta Cryst.* E**76**, 1–11. 10.1107/S2056989019016244 PMC6944088 31921444

[bb21] Sun, Y.-X., Zhang, R., Jin, Q.-M., Zhi, X.-J. & Lü, X.-M. (2006). *Acta Cryst.* C**62**, o467–o469. 10.1107/S0108270106021512 16891721

[bb22] Tan, S. L., Jotani, M. M. & Tiekink, E. R. T. (2019). *Acta Cryst.* E**75**, 308–318. 10.1107/S2056989019001129 PMC6399703 30867939

[bb23] Westrip, S. P. (2010). *J. Appl. Cryst.* **43**, 920–925.

[bb24] Yan, G.-B., Zheng, Y.-F., Zhang, C.-N. & Yang, M.-H. (2006). *Acta Cryst.* E**62**, o5328–o5329.

[bb25] Zyss, J. (1979). *J. Chem. Phys.* **70**, 3341–3349.

