# Supplementary material for: Syntheses, crystal structures, Hirshfeld surface analyses and energy frameworks of two 4-amino­anti­pyrine Schiff base compounds: (E)-4-{[4-(di­ethyl­amino)­benzyl­idene]amino}-1,5-dimethyl-2-phenyl-1H-pyrazol-3(2H)-one and (E)-4-[(4-fluoro­benzyl­idene)amino]-1,5-dimethyl-2-phenyl-1H-pyrazol-3(2H)-one
Source: Acta Crystallogr E Crystallogr Commun. 2023 May 12;79(Pt 6):538–44. doi: 10.1107/S2056989023004085 (PMC10242735; doi:10.1107/S2056989023004085)
Supplement: Supplementary file 6 [file e-79-00538-sup6.pdf]

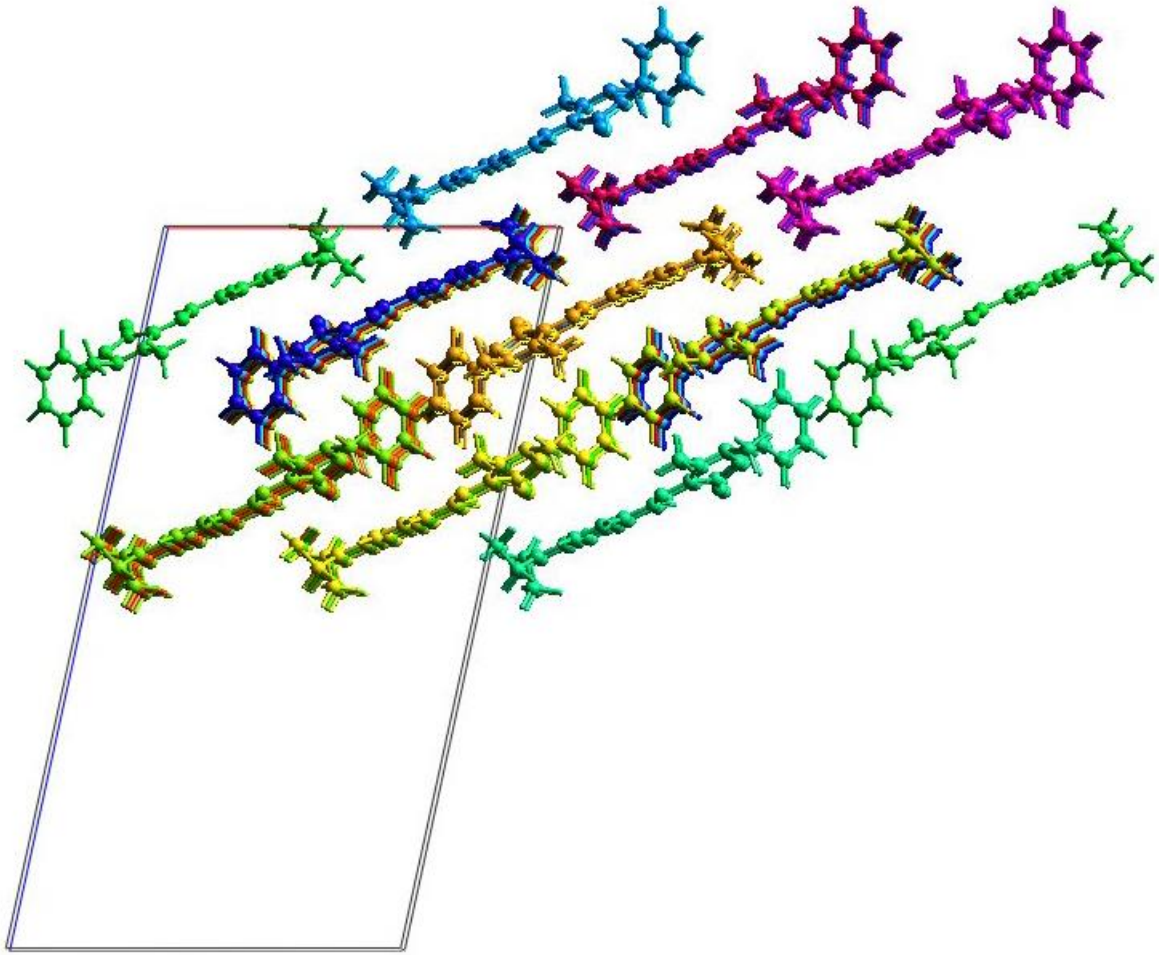

	N	Symop	R	Electron Density	E_ele	E_pol	E_dis	E_rep	E_tot
	2	x+1/2, y+1/2, z	9.28	HF/3-21G	-8.2	-6.4	-34.6	18.4	-28.8
	2	-x+1/2, y+1/2, -z+1/2	15.85	HF/3-21G	-8.3	-1.5	-18.9	0.0	-26.5
	2	x, y, z	7.09	HF/3-21G	-17.6	-7.9	-40.7	24.3	-40.1
	2	-x, y, -z+1/2	11.45	HF/3-21G	-3.6	-1.0	-8.8	3.1	-9.7
	2	x+1/2, y+1/2, z	13.67	HF/3-21G	1.1	-0.1	-0.6	0.0	0.5
	2	-x+1/2, y+1/2, -z+1/2	18.75	HF/3-21G	0.4	-0.0	-0.3	0.0	0.1
	1	-x, y, -z+1/2	8.99	HF/3-21G	-7.2	-3.3	-65.5	38.0	-37.6
	2	x, y, z	17.16	HF/3-21G	-0.5	-0.0	-0.9	0.0	-1.3
	2	-x+1/2, y+1/2, -z+1/2	9.10	HF/3-21G	1.0	-0.2	-2.2	0.0	-1.1
	1	-x, -y, -z	9.28	HF/3-21G	3.5	-0.4	-2.8	0.0	0.7
	2	x+1/2, y+1/2, z	9.28	HF/3-21G	-12.0	-5.0	-49.4	21.4	-42.6
	1	-x, -y, -z	8.82	HF/3-21G	1.6	-0.3	-2.3	0.0	-0.7
	1	-x+1/2, -y+1/2, -z	10.85	HF/3-21G	-8.8	-4.9	-45.4	18.2	-38.3
	2	x+1/2, y+1/2, z	13.67	HF/3-21G	0.6	-0.1	-1.3	0.0	-0.6
	1	-x, -y, -z	18.17	HF/3-21G	19.3	-0.6	-22.9	0.0	-1.4
	1	-x+1/2, -y+1/2, -z	13.28	HF/3-21G	-3.1	-0.8	-16.2	0.0	-18.3
	1	-x, -y, -z	20.95	HF/3-21G	-0.1	-0.0	-0.5	0.0	-0.6
	1	-x, -y, -z	17.94	HF/3-21G	1.6	-0.2	-6.7	0.0	-4.6
	1	-x+1/2, -y+1/2, -z	12.64	HF/3-21G	-0.1	-0.1	-1.7	0.0	-1.8

Fig. S1 The colour-coded interaction mapping within a radius of 6 Å of a central reference molecule and the various contributions to the total energy (E_{tot}) for compound **I**.

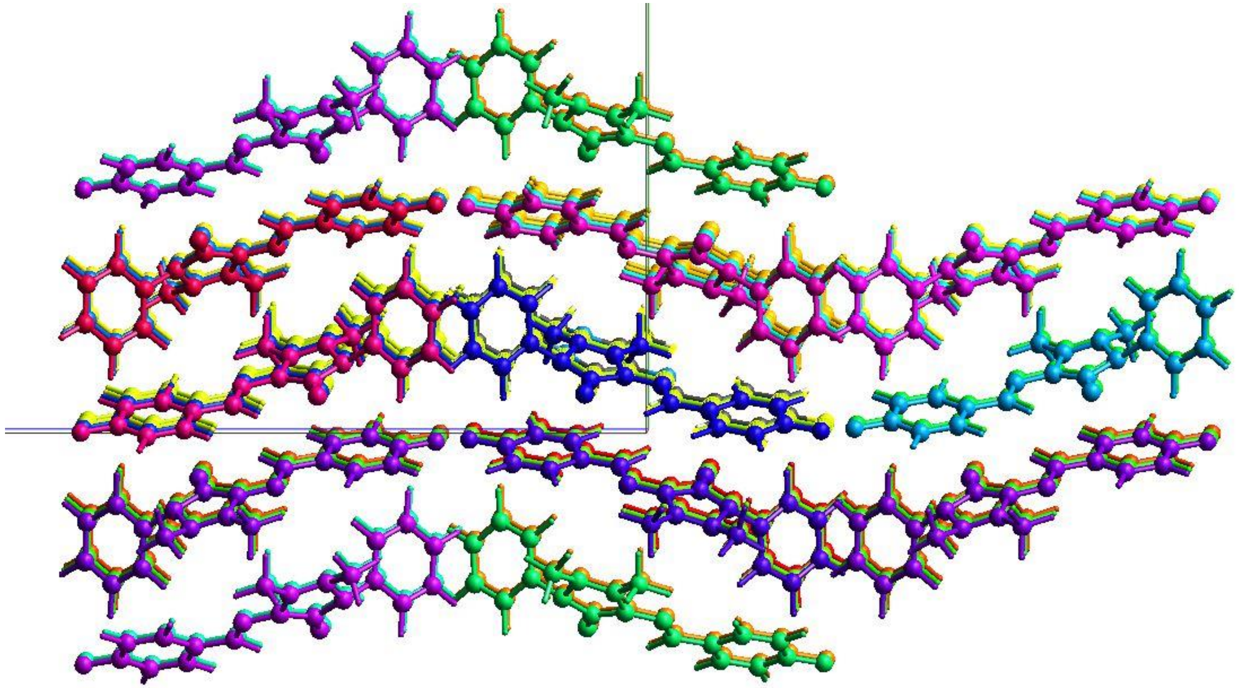

	N	Symop	R	Electron Density	E_ele	E_pol	E_dis	E_rep	E_tot
	1	-x, -y, -z	4.63	HF/3-21G	-7.7	-6.1	-88.6	54.5	-47.5
	1	-x+1/2, -y, z+1/2	14.33	HF/3-21G	-1.6	-0.7	-5.8	0.0	-7.4
	1	-x+1/2, y+1/2, z	8.60	HF/3-21G	-0.4	-0.4	-2.6	0.0	-3.0
	1	x+1/2, -y+1/2, -z	5.36	HF/3-21G	-9.4	-5.2	-54.5	31.0	-36.9
	0	x, -y+1/2, z+1/2	14.05	HF/3-21G	-1.2	-0.4	-6.3	0.0	-7.2
	1	x+1/2, y, -z+1/2	12.74	HF/3-21G	15.0	-1.9	-20.4	0.0	-4.4
	1	x, y, z	6.79	HF/3-21G	-19.3	-6.7	-30.3	13.0	-40.7
	0	-x, -y, -z	7.22	HF/3-21G	-21.5	-12.8	-29.2	17.9	-42.1
	0	-x+1/2, -y, z+1/2	16.80	HF/3-21G	0.8	-0.0	-0.3	0.0	0.5
	1	x+1/2, y, -z+1/2	15.06	HF/3-21G	-3.3	-1.1	-5.6	0.0	-9.1
	0	-x+1/2, y+1/2, z	12.28	HF/3-21G	-0.1	-0.0	-0.3	0.0	-0.4
	0	-x, y+1/2, -z+1/2	15.86	HF/3-21G	-0.7	-0.0	-0.3	0.0	-1.0
	1	x+1/2, -y+1/2, -z	11.00	HF/3-21G	-2.7	-0.2	-1.0	0.0	-3.8
	0	x, -y+1/2, z+1/2	15.61	HF/3-21G	-0.9	-0.1	-0.6	0.0	-1.5
	1	x+1/2, y, -z+1/2	17.86	HF/3-21G	-0.2	-0.0	-0.1	0.0	-0.3
	1	x, -y+1/2, z+1/2	15.61	HF/3-21G	1.0	-0.0	-0.4	0.0	0.6
	1	x+1/2, y, -z+1/2	15.95	HF/3-21G	0.8	-0.1	-0.3	0.0	0.5
	1	x, y, z	13.58	HF/3-21G	-1.4	-0.1	-0.2	0.0	-1.7
	0	-x, -y, -z	13.23	HF/3-21G	-0.3	-0.0	-0.3	0.0	-0.5
	0	-x+1/2, -y, z+1/2	21.24	HF/3-21G	0.2	-0.0	-0.0	0.0	0.2
	0	-x, y+1/2, -z+1/2	19.35	HF/3-21G	-0.3	-0.0	-0.0	0.0	-0.3
	0	x, -y+1/2, z+1/2	19.54	HF/3-21G	-0.7	-0.0	-0.1	0.0	-0.8
	0	x+1/2, -y+1/2, -z	17.47	HF/3-21G	-0.8	-0.0	-0.0	0.0	-0.9
	1	x+1/2, y, -z+1/2	20.95	HF/3-21G	-0.0	-0.0	-0.0	0.0	-0.1
	1	x, -y+1/2, z+1/2	19.54	HF/3-21G	0.4	-0.0	-0.0	0.0	0.4

Fig. S2 The colour-coded interaction mapping within a radius of 6 Å of a central reference molecule and the various contributions to the total energy (E_{tot}) for compound **II**.

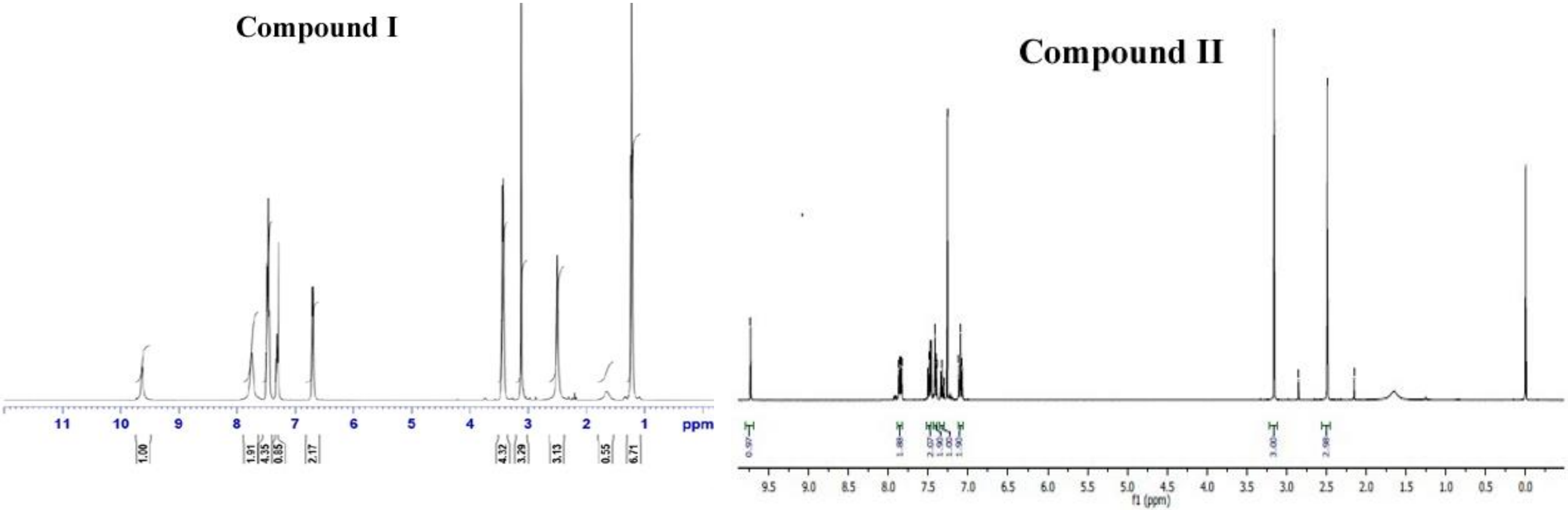

Fig. S3 ^{1}H NMR Spectra

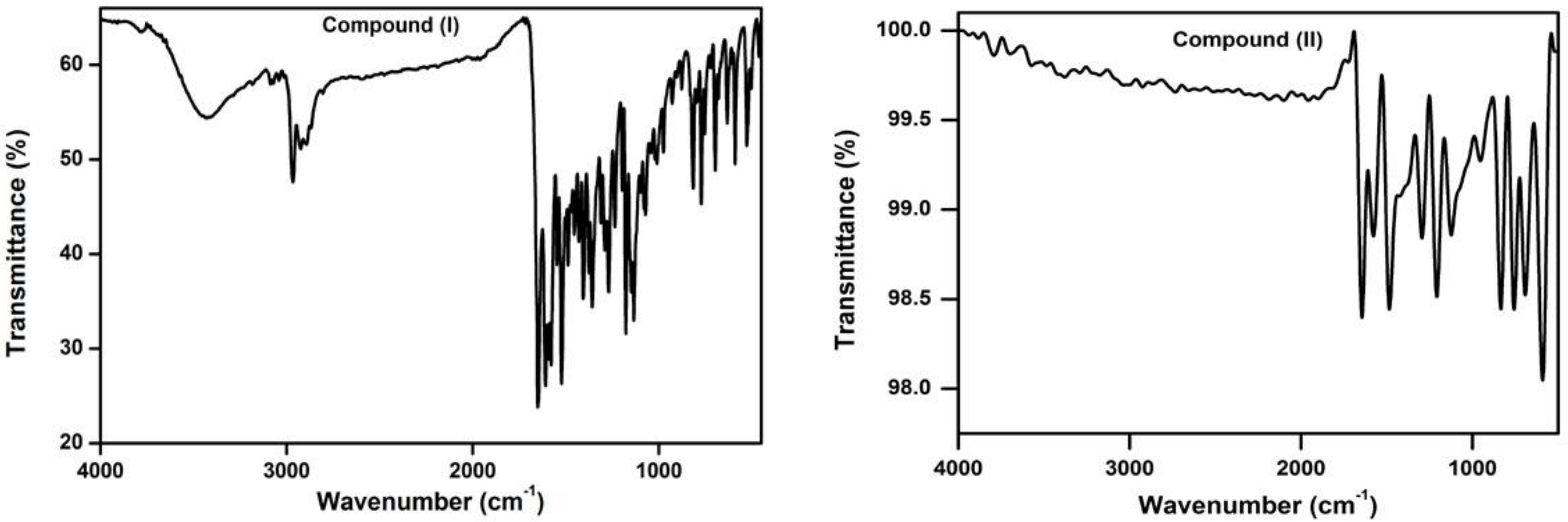

Fig. S4 FT-IR Spectra
